# Feasibility, acceptability, and short-term impact of a brief sexually transmitted infection intervention targeting U.S. Military personnel and family members

**DOI:** 10.1186/s12889-022-13096-x

**Published:** 2022-04-02

**Authors:** Anjali Kunz, Amber Moodley, Donn J. Colby, Michele Soltis, Wesley Robb-McGrath, Alexandra Fairchok, Paul Faestel, Amanda Jungels, Alexis A. Bender, Edwin Kamau, Gina Wingood, Ralph DiClemente, Paul Scott

**Affiliations:** 1 Madigan Army Medical Center, Joint-Base Lewis McChord, Tacoma, WA US; 2 Henry M. Jackson Foundation for the Advancement of Military Medicine, Bethesda, MD US; 3 United States Military HIV Research Program, Walter Reed Army Institute of Research, Silver Spring, MD US; 4 Behavioral and Social Health Outcomes Program, Army Public Health Center, Edgewood, MD US; 5 Center for Teaching Excellence, Rice University, Houston, TX US; 6 Emory University School of Medicine, Atlanta, GA US; 7 Columbia University Mailman School of Public Health, New York, NY US; 8 New York University School of Global Public Health, New York, NY US; 9 Emerging Infectious Diseases Branch, Walter Reed Army Institute of Research, Silver Spring, MD US

**Keywords:** Military, Evidence-based interventions, Prevention, Sexually transmitted infections, Human immunodeficiency virus

## Abstract

**Background:**

Over the past 10 years, incidence of sexually transmitted infections (STIs) has increased to record numbers in the United States, with the most significant increases observed among adolescents and young adults. The US military, where the majority of active duty personnel are 18–30 years old, has seen similar increases. However, the US military does not yet have a standardized, service-wide program for STI education and prevention.

**Methods:**

The KISS intervention (Knocking out Infections through Safer-sex and Screening) was adapted from an evidence-based intervention endorsed by the US Centers for Disease Control and Prevention and consisted of a one-time, small group session. Content included STI/HIV knowledge and prevention, condom use skills, and interpersonal communication techniques. The intervention was pilot tested for feasibility and acceptability among a population of service members and medical beneficiaries at Joint Base Lewis-McChord in Washington state.

**Results:**

A total of 79 participants aged 18–30 years were consented to participate in the pilot study and met entry criteria, 66/79 (82.5%) attended the intervention session, and 46/66 (69.7%) returned at 3 months for the final follow-up assessment. The intervention sessions included 31 male (47.0%) and 35 female (53.0%) participants. Almost all participants felt comfortable discussing sexual issues in the group sessions, reported that they intended to practice safer sex after the intervention, and would also recommend the intervention to friends. Knowledge about STI/HIV prevention significantly increased after the intervention, and intervention effects were maintained at 3 months. About one-fifth of participants tested positive for N. gonorrhea or C. trachomatis infection at enrollment, while none had recurrent STIs at the final visit. Use of both male and female condoms increased after the intervention.

**Conclusions:**

The KISS intervention was feasible to implement in the military setting and was acceptable to the active duty service members and other medical beneficiaries who participated in the pilot project. Further studies are needed to determine if the KISS intervention, or others, effectively decrease STI incidence in active duty personnel and would be appropriate for more widespread implementation.

**Trial Registration:**

Retrospectively registered as the pilot phase of clinicaltrials.gov NCT04547413, “Prospective Cohort Trial to Assess Acceptability and Efficacy of an Adapted STI/HIV Intervention Behavioral Intervention Program in a Population of US Army Personnel and Their Medical Beneficiaries—Execution Phase.”

**Supplementary Information:**

The online version contains supplementary material available at 10.1186/s12889-022-13096-x.

## Introduction

Increasing rates of sexually transmitted infections (STIs) among active duty service members is of great concern, especially since STI rates in the U.S. military have historically been higher than their civilian counterparts [1–4]. Nearly half (46%) of active duty U.S. military personnel are 25 years old or younger, making them especially high-risk for acquiring STIs [5]. Increasing rates of STIs directly affect the costs of services provided through the Military Health System, and indirectly affect personnel productivity and troop readiness when service members have symptomatic infections that require medical testing and treatment [6].

Behaviors that enhance risk for STIs are common in the military population, including binge drinking, inconsistent condom use, and multiple sexual partnerships [7–9]. STI rates are consistently higher among service members that are younger, unmarried, female, non-Hispanic black, of lower rank, and with lower educational achievement [10]. Less is known about risky sexual behaviors in military dependents and beneficiaries, leaving the relationship between sexual behaviors and STIs among this military subset unclear.

As part of a comprehensive approach to combat increasing rates of STIs and HIV in the U.S., the Centers for Disease Prevention and Control (CDC) has advocated for increased testing, screening, and treatment for STIs and HIV, along with STI/HIV evidence-based behavioral intervention (EBI) programs that target reduction in risk behaviors and provide skills and resources to assist behavioral change [11–14].

The simple adaptation of a civilian behavioral intervention program may not be successful, given the complexity, mobility, and diversity of the U.S. military population [6]. A recent review spanning 1991–2018 found a total of 8 published reports on interventions designed to decrease STIs among US military personnel, only 3 of which included STI incidence as an outcome measure [15]. Of these, only one study, published in 2005 and conducted with only female marine recruits on a single military base, demonstrated intervention efficacy in decreasing incident STIs [16].

Although STI/HIV prevention education is mandated for all U.S. service members, there is neither a standardized curriculum nor defined qualifications for personnel to conduct the training. Currently, most STI/HIV education is delivered through didactic sessions rather than programs that target attitudes, beliefs, and behaviors prevalent in the military community.

There remains a compelling need to develop evidence-based behavioral intervention programs designed to modify high-risk sexual behaviors that increase the risk for STI/HIV among high-risk members of the military community. To address this need, a pilot intervention study was implemented at the Joint Base Lewis-McChord (JBLM) military base to determine the feasibility and acceptability of an adapted, evidence-based STI/HIV behavioral intervention program in a population of US Army personnel and their medical beneficiaries.

## Methods

The pilot study enrolled a single group with a pre-test and post-test design and completed recruitment and study visits from October 2016 to May 2017. The pilot was designed to demonstrate feasibility of implementing the intervention in the military setting, and acceptability among military personnel. Therefore, the pilot did not include a control group and was not powered to show efficacy of clinical outcomes. The efficacy assessment of the intervention is ongoing with results expected in 2023 (Clinicaltrials.gov registration NCT04547413).

Participants were 18–30 years old, Army active duty or a medical beneficiary (most commonly the spouse of an active duty soldier), HIV negative at enrollment, not pregnant or trying to conceive, and not scheduled to be deployed within at least 3 months from enrolment. Recruitment occurred at the Madigan Army Medical Center (MAMC) Preventive Medicine clinic (PMC), located on the JBLM military base near Tacoma, Washington, United States, which provides walk-in services for patients with suspected STIs.

A member of the research staff approached potential research participants in the clinic to introduce the study and invite them to screen for enrollment immediately or at a later date. All participants provided written informed consent. Clinical samples and data were de-identified for analyses. The institutional review boards at Walter Reed Army Institute for Research (WRAIR) and the Western Regional Medical Command approved the study.

All study participants enrolled in the pilot study were invited to 3 study visits over three to four months. Visit 1 (1-h in duration) included the informed consent process and laboratory testing completed as part of routine care if STI/HIV testing had not been completed in the previous 90 days. Clinical sampling included diagnostic testing for *Chlamydia trachomatis* (chlamydia), *Neisseria gonorrhea* (gonorrhea), and HIV. Participants diagnosed with bacterial STIs were promptly treated and provided standard-of-care prevention counseling. Visit 2 (4 h in duration) involved administration of the intervention, a sexual health group class described in more detail below. At visit 3 (1 h), scheduled three months after visit 2, participants returned to complete follow-up clinical testing for STI/HIV and repeat questionnaires. The study questionnaires included a Sexual Risk Assessment (SRA) and an STI/HIV knowledge assessment scale completed at visits 2 and 3. A feedback form regarding the intervention was completed at the end of visit 2. In the study questionnaires, sex was defined as any vaginal, oral, or anal penetration.

Visit 1 (screening and enrolment) and visit 3 (post-intervention follow-up) were conducted at the MAMC Preventive Medicine Clinic (PMC). The visit 2 intervention session took place on weekends and active duty participants were required to be on-leave or off-duty to receive compensation at this visit. Participants received an incentive ($25 for visits 1 and 3, $75 for visit 2) as remuneration for taking time to participate in the study. In addition, participants could receive an additional $50 to offset childcare expenses for the visit 2 intervention session.

### Adaptation of the intervention for a military population

The intervention, Knocking-out Infections through Safer-sex and Screening (KISS), was a single, interactive group session administered in groups of 5 to 8 participants of the same gender, delivered by a trained study health educator on the research team. The intervention aimed to decrease STI incidence through increased condom usage.

The intervention was adapted for use in the military from HORIZONS, a US Centers for Disease Control and Prevention (CDC)-defined evidence-based intervention [17]. HORIZONS is based on Social Cognitive Theory and the Theory of Gender and Power and includes content on STI/HIV prevention knowledge, condom use skills, interpersonal communication techniques, and perceived peer norms supportive of condom use and regular STI/HIV screening [18]. The HORIZONS intervention has demonstrated efficacy in increasing condom usage and decreasing STI incidence in a population of young African American women.

We used the ADAPT-ITT framework to modify the intervention for relevance across the diversity of gender, race, and ethnic groups in the American military population [19]. Vignettes were developed to represent different racial and ethnic identities, as well as multi-racial and -ethnic personnel. Significant content changes included a greater focus on the threats posed by substance use and intimate partner violence for STI acquisition and transmission. These components of KISS were amplified as evidence suggests a high prevalence of alcohol and other drug use, as well as coercive sex, in military populations [20, 21].

### Visit 1: Consenting, screening, and biological testing

Visit 1 was approximately an hour. Participants were informed that they would be asked to complete three visits over three months and attend one weekend interactive group session regarding sexual health being evaluated for feasibility of implementation in the military. After informed consent was obtained, participants provided a urine specimen for chlamydia and gonorrhea (CT/NG) using nucleic acid amplification technology (NAAT) and a blood sample for HIV using ELISA/WB following standard clinic procedures. Clinical testing was not repeated if it had already been performed within the previous 90 days and test results were available in the electronic medical record.

### Visit 2: Sexual health education class at the preventive medicine clinic

Visit 2 was about four hours and took place in a conference room at the PMC. There were separate groups for men and women. Participants only used their first names and were asked to wear plain civilian clothes to help maintain confidentiality. This was considered especially important in the military setting due to rank and the effect that it may have on interpersonal interactions. If two or more participants in a visit 2 group session knew each other (e.g., more than one person from the same unit), they were given the option to change to a different group on a different day or withdraw from the study.

Prior to the sexual health group session, participants completed the SRA survey (Additional file 1) and a 30-item STI/HIV knowledge assessment (Additional file 2). Immediately after the session, participants repeated the STI/HIV knowledge assessment and a feedback form (Additional file 3, Fig. 3) to identify the most useful aspects of the group session. Study participants received information on protecting their sexual health and skills training on use of barrier protection. The group sessions took place on weekends to ensure active duty personnel would be off-duty and able to participate and receive compensation per Department of Defense regulations.

### Visit 3: Follow-up at the preventive medicine clinic

This visit was about one hour and was scheduled three months after the intervention session. Participants returned to repeat the SRA survey, STI/HIV knowledge assessment, and STI and HIV testing. Visit 3 occurred on weekdays during regular clinic hours.

### Statistical analysis

Participants who completed both visit 2 and visit 3 and had available data (non-missing values) for a particular variable were included in analyses comparing the pre- and post-intervention time points. We used descriptive measures to summarize data. Continuous variables were summarized with median and interquartile range (IQR); categorical variables were summarized with frequency (%); comparisons were made with the Fisher exact test. All p values are two-sided and a p-value of less than 0.05 was considered significant. Statistical analyses and graphics were done using GraphPad Prism 8.2.0 and R package.

## Results

**Fig. 1:**
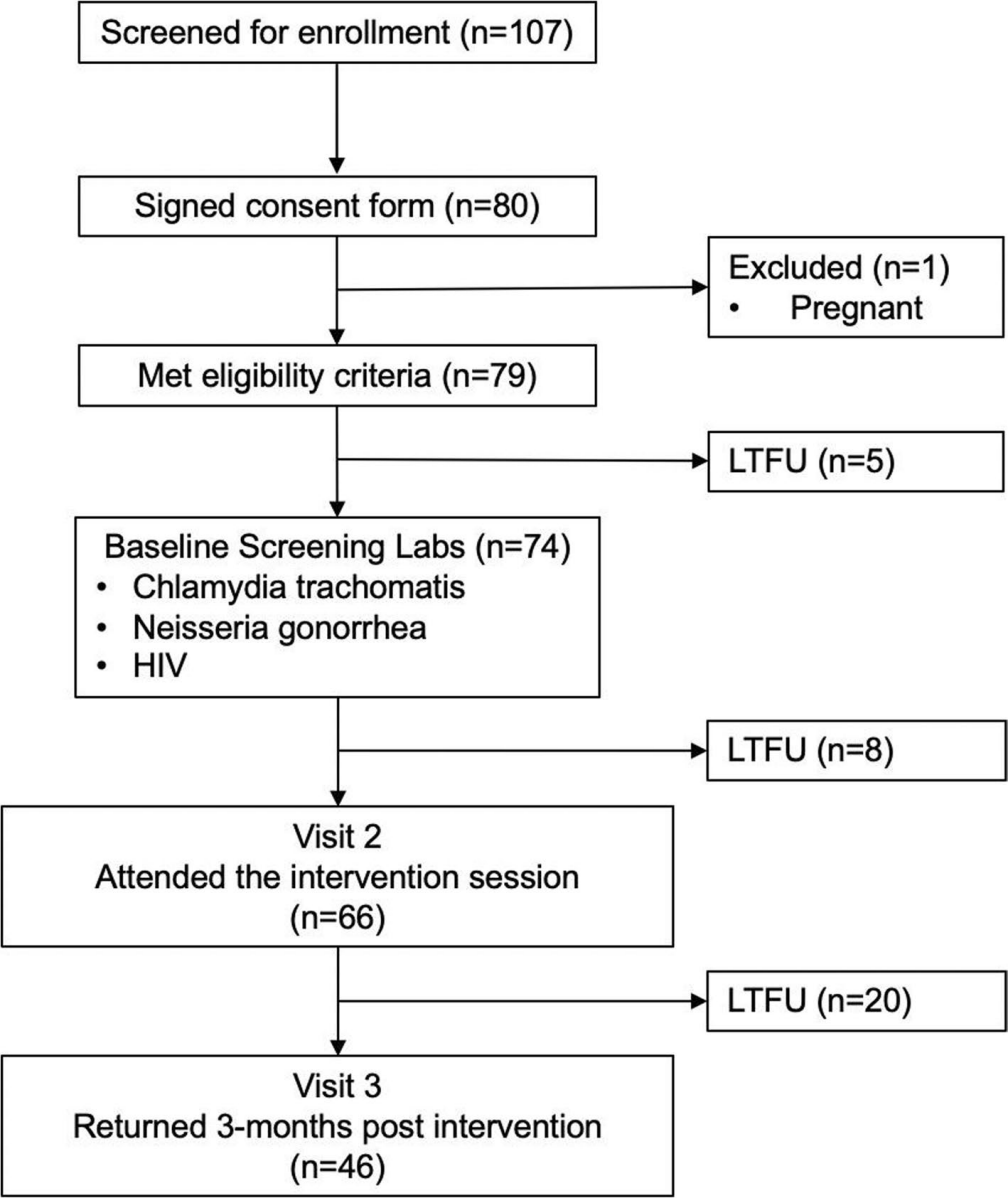
Flowchart of study screening, enrolment, and follow-up

A total of 80 participants aged 18–30 years consented to participate in the pilot study, of whom 79 met entry criteria, 66/79 (83.5%) returned to visit 2, and 46/66 (69.7%) returned 3 months later for the third and final follow-up visit (Fig. 1). All 66 participants who attended the intervention session at visit 2 were included in the data analysis; demographic data are presented in Table 1. The study population included 31 male (47.0%) and 35 female (53.0%) participants, 18 (27.3%) of whom identified as White/Caucasian, 18 (27.3%) as Black/African American, 9 (13.6%) as Hispanic/Latino, 2 (3.0%) as Asian/Pacific Islander and 19 (28.8%) identified as other or multiracial.

**Table 1:** Demographic characteristics

Item	Overall (*n* = 66)		Male (*n* = 31)		Female (*n* = 35)	
	N	%	N	%	N	%
**Gender** (n, %)	66	100%	31	47.0%	35	53.0%
**Median years of military service** (IQR)	2	(1—4)	2	(1—4)	3	(2—6)
**Education** (n, %)						
High school diploma or GED	22	33.3%	15	48.4%	7	20.0%
Some College	27	40.9%	9	29.0%	18	51.4%
Associates degree	7	10.6%	4	12.9%	3	8.6%
Bachelor's degree	3	4.5%	1	3.2%	2	5.7%
Vocational/technical school (other than military)	4	6.1%	1	3.2%	3	8.6%
Completed graduate/professional degree	3	4.5%	1	3.2%	2	5.7%
**Race/Ethnicity** (n, %)						
White or Caucasian	18	27.3%	9	29.0%	9	25.7%
Black or African American	18	27.3%	7	22.6%	11	31.4%
Hispanic or Latino	9	13.6%	5	16.1%	4	11.4%
Asian	2	3.0%	2	6.5%	0	0.0%
Other	19	28.8%	8	25.8%	11	31.4%
**Current marital status** (n, %)						
Single	29	43.9%	18	58.1%	11	31.4%
In a committed relationship	6	9.1%	2	6.5%	4	11.4%
Married	20	30.3%	6	19.4%	14	40.0%
Separated/Divorced	10	15.2%	4	12.9%	6	17.1%
Other	1	1.5%	1	3.2%	0	0.0%
**Relationship status changed in the last 12 months** (n, %)						
No	38	57.6%	17	54.8%	21	60.0%
Yes	24	36.4%	11	35.5%	13	37.1%
Prefer not to answer	4	6.1%	3	9.7%	1	2.9%
**Service Branch** (n, %)						
Army	53	80.3%	30	96.8%	23	65.7%
Beneficiary	13	19.7%	1	3.2%	12	34.3%
** Total months away from home/permanent duty station(s)** (Median, IQR)	2	(0—6.75)	2	(0—7.5)	1	(0—4)
** Total times been away from home/permanent duty station(s)** (Median, IQR)	1.5	(0—4)	1.5	(0—4)	1.5	(0—4.25)

With respect to sexual risk behavior reported at visit 2 (Table 2), the majority (89.4%) reported having sex in the last three months; 66.7% reported having sex with one partner and 22.7% reported having sex with two or more partners. Most (75.8%) reported having vaginal sex with a spouse or regular partner, 30.3% reported sex with an occasional partner and 10.6% reported sex with a one-time partner or sex worker in the past 3 months. Self-reported sexual orientation was 83.3% heterosexual, 10.6% bisexual and 3.0% “not sure.”

**Table 2:** Sexual history and sexually transmitted Infections (visit 2)

Item	Overall (*n* = 66)		Male (*n* = 31)		Female (*n* = 35)	
	N	%	N	%	N	%
**Sexually transmitted infections** (n, %)						
Chlamydia (positive result)	8	12.1%	6	19.4%	2	5.7%
Gonorrhea (positive result)	4	6.1%	2	6.5%	2	5.7%
Testing not performed	2	3.0%	0	0.0%	2	5.7%
**Number of lifetime sexual partners** (n, %)						
1–10	35	53.0%	11	35.5%	24	68.6%
11–20	17	25.8%	10	32.3%	7	20.0%
≥21	9	13.6%	7	22.6%	2	5.7%
Prefer not to answer	5	7.6%	3	9.7%	2	5.7%
** How many times did you have sex in the past month?** (median, IQR)	3	(1—7.5)	4	(1.5—10)	2	(0—5)
** How many times did you have sex in the past 3 months?** (median, IQR)	10	(4- 20)	14	(4—30)	6	(4—15)
**Number of sexual partners in the past 3 months** (n, %)						
One partner	44	66.7%	19	61.3%	25	71.4%
More than one partner	15	22.7%	8	25.8%	7	20.0%
No sex in past 3 months	7	10.6%	4	12.9%	3	8.6%
**Vaginal Sex with which type of partner in past 3 months** (n, %)						
Spouse or main partner	50	75.8%	22	71.0%	28	80.0%
Occasional partner	20	30.3%	10	32.3%	10	28.6%
Sex Worker or one-time partner	7	10.6%	6	19.4%	1	2.9%
**Oral Sex with which type of partner in past 3 months** (n, %)						
Spouse or main partner	47	71.2%	21	67.7%	26	74.3%
Occasional partner	17	25.8%	10	32.3%	7	20.0%
Sex Worker or one-time partner	5	7.6%	4	12.9%	1	2.9%
**Anal Sex with which type of partner in past 3 months** (n, %)						
Spouse or main partner	5	7.6%	2	6.5%	3	8.6%
Occasional partner	2	3.0%	1	3.2%	1	2.9%
Sex Worker or one-time partner	0	0.0%	0	0.0%	0	0.0%
**How many new sexual partners have you had in the last 3 months?** (median, IQR)						
Males	0	(0—1)	0	(0—0)	0	(0—1)
Females	0	(0—2)	1	(0 – 3)	0	(0—0)
**During the past 3 months did you engage in vaginal, oral, or anal sex with more than one person at the same time?** (n, %)						
No	59	89.4%	26	83.9%	33	94.3%
Yes	7	10.6%	5	16.1%	2	5.7%
**Past sexual partners** (n, %)						
Men	28	42.4%	1	3.2%	27	77.1%
Women	30	45.5%	29	93.5%	1	2.9%
Both	7	10.6%	0	0.0%	7	20.0%
I have never had sex	1	1.5%	1	3.2%	0	0.0%
**How do you self-identify?** (n, %)						
Heterosexual or straight	55	83.3%	29	93.5%	26	74.3%
Homosexual or gay	0	0.0%	0	0.0%	0	0.0%
Bisexual	7	10.6%	1	3.2%	6	17.1%
Something else	1	1.5%	0	0.0%	1	2.9%
Not sure	2	3.0%	0	0.0%	2	5.7%
Prefer not to answer	1	1.5%	1	3.2%	0	0.0%
**Frequency of alcohol consumption** (n, %)						
Daily	3	4.5%	3	9.7%	0	0.0%
Weekends only	10	15.2%	4	12.9%	6	17.1%
4 or more times a week	1	1.5%	0	0.0%	1	2.9%
2–3 times a week	7	10.6%	5	16.1%	2	5.7%
2–4 times a month	14	21.2%	8	25.8%	6	17.1%
Monthly or less	19	28.8%	7	22.6%	12	34.3%
Never	12	18.2%	4	12.9%	8	22.9%
**Total number of drinks consumed on typical drinking day** (n, %)						
1 or 2	25	37.9%	11	35.5%	14	40.0%
3 or 4	21	31.8%	11	35.5%	10	28.6%
5 or 6	10	15.2%	3	9.7%	7	20.0%
7 or 9	1	1.5%	1	3.2%	0	0.0%
10 or more	1	1.5%	1	3.2%	0	0.0%
Prefer not to answer	8	12.1%	4	12.9%	4	11.4%

**Fig. 2:**
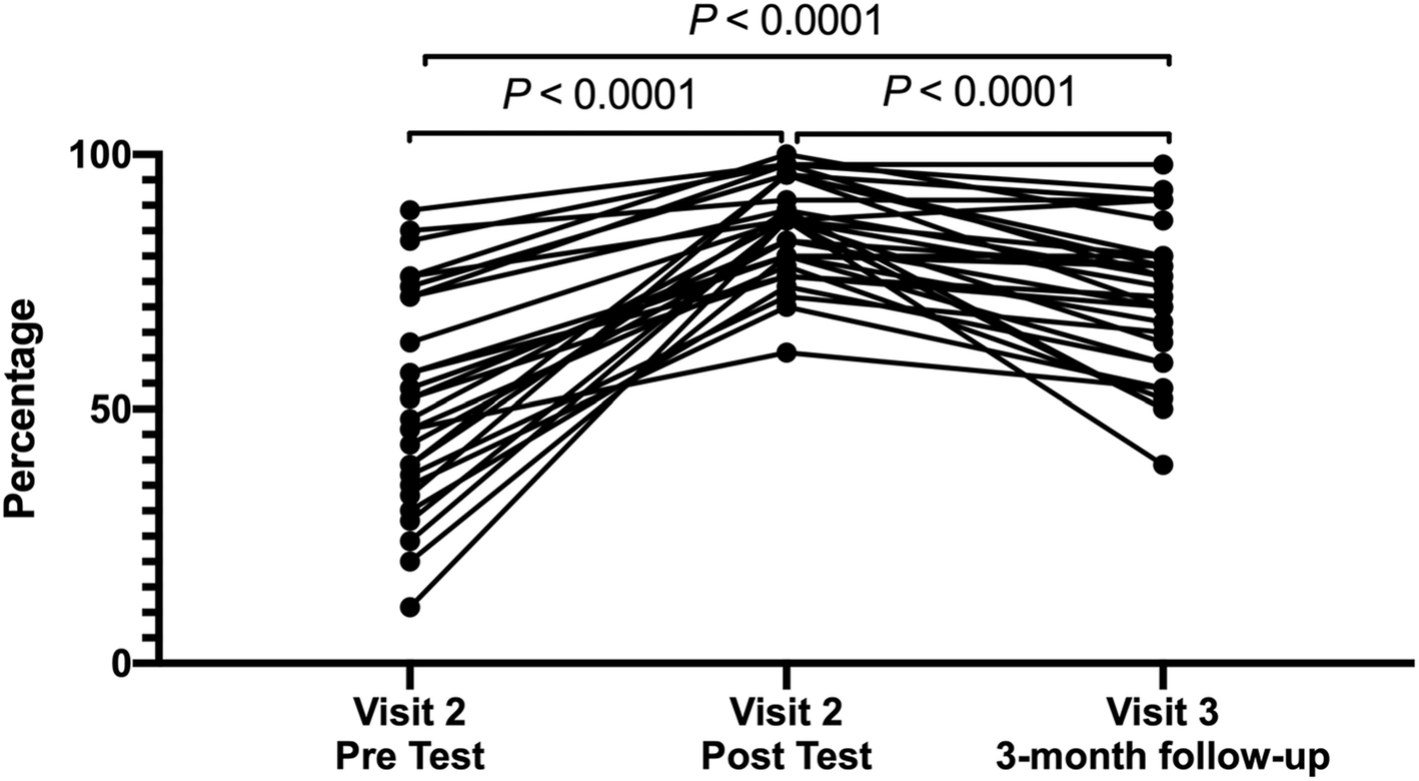
STI/HIV Knowledge Assessments: percent correct responses to each question at 3 time points (visit 2 *n* = 66, visit 3 *n* = 46)

The STI/HIV Knowledge Assessment scale was administered twice: at visit 2, as a pre-test prior to the intervention session and a post-test immediately afterward. The same assessment was administered again at visit 3, approximately 90 days later. As presented in Fig. 2, participants provided an average of 52.2% correct responses at the visit 2 pre-test, 87.0% correct at the visit 2 post-test, and 72.8% correct responses at study visit 3 (all *p* < 0.0001). On average, 32.7% of participants responded that they did not know the correct answer for each question at the visit 2 pre-test (Range 6.5%—67.4%) compared to an average of 12.5% (Range 2.2%—26.1%) at visit 3.

**Fig. 3:**
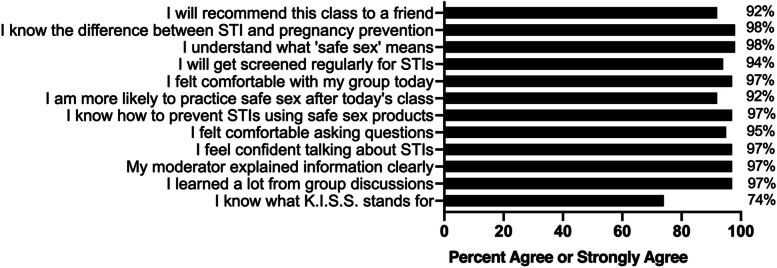
Results of the KISS Intervention Feedback Survey (*n* = 66)

A participant feedback form was administered directly after the interactive group session to assess participants’ acceptability of the intervention and to provide the instructor with feedback on the quality and delivery of the intervention session. Overall, the feedback illustrated that participants felt the intervention was acceptable and well-delivered (Fig. 3).

### STIs and condom use

At the baseline screening visit (visit 1), 74 out of the 79 participants who enrolled completed chlamydia, gonorrhea and HIV antibody testing. At least one positive STI test was detected in 18.9% (14/74) of participants, with 11 (14.9%) positive chlamydia tests, 5 (6.8%) positive gonorrhea tests, and two participants testing positive for both. The prevalence of STIs at baseline was not different among the 46 participants who completed all study visits than among the 28 who did not attend visit 3 (17.4% vs. 21.4%, *p* = 0.89).

Among the 66 participants who provided demographic data, there were 12 STIs among 11 (16.7%) participants. Participants with STIs were more likely to be male (7/11), active duty (11/11), single or separated (11/11), and black or Hispanic (7/11) than the cohort as a whole.

No study participants tested positive for HIV antibodies at the baseline screening visit. At visit 3, 46 participants returned for clinical testing; none tested positive for an STI or HIV.

**Fig. 4:**
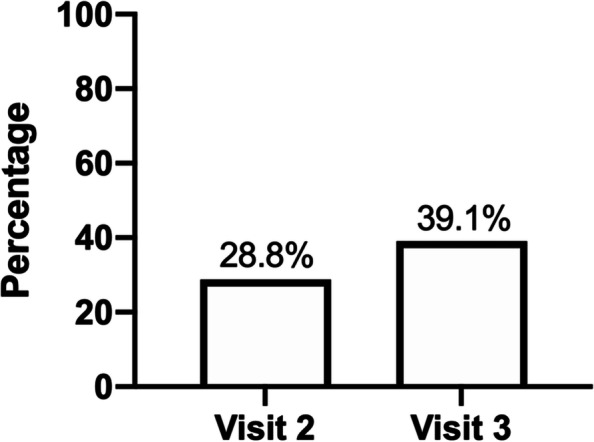
Condom use at last sexual encounter (visit 2 *n* = 66, visit 3 *n* = 46)

**Fig. 5:**
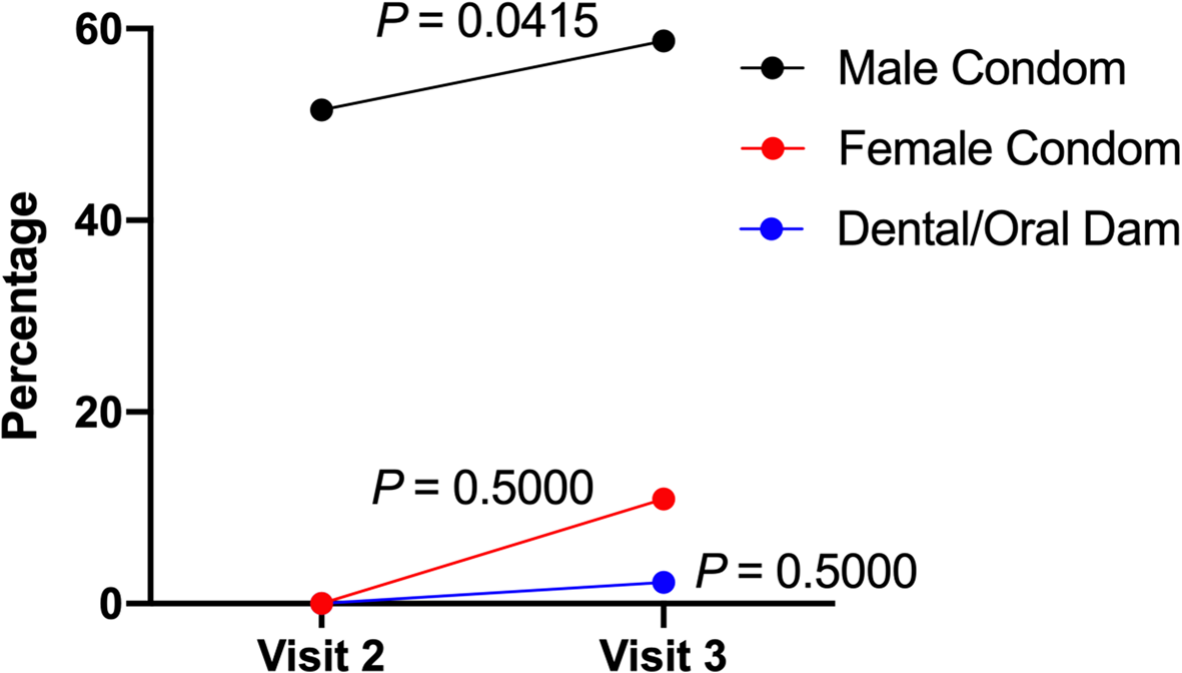
Barrier protection use in past 3 months (visit 2 *n* = 66, visit 3 *n* = 46)

Prior to the KISS intervention, 28.8% of participants reported using a condom at their last sexual encounter. At the final visit this percentage increased to 39.1% (*p* < 0.0001) (Fig. 4). Of the 27 participants at visit 3 who reported having vaginal sex with either an occasional partner, sex worker, or one-time partner, condom use was reported as 33.4% always, 48.1% sometimes, and 18.5% never. Additionally, an increase was observed in the proportion reporting male condom use in the past three months, as well as increases in the use of other protective barrier methods (Fig. 5). Specifically, before participating in the KISS group session, 51.5% of participants reported male condom use in the past three months. At the visit 3 follow-up, this increased to 58.7%. Female condom use increased from none reported at visit 2 to 10.9% at Visit 3.

No significant protocol deviations or adverse events related to study participation were reported.

## Discussion

This pilot study demonstrated that the KISS intervention, designed to address the sexual health needs of a military population, was both feasible to implement and acceptable to the target population. Furthermore, there were encouraging signs that active duty military personnel and their medical beneficiaries had better knowledge and used condoms more frequently after attending the intervention session.

In the post-intervention feedback forms, almost all participants reported that they felt comfortable asking questions and discussing STIs, and that they learned a lot during the intervention. Moreover, 92% reported that they were more likely to practice safer sexual behaviors after the class and that they would recommend the intervention to a friend.

The KISS intervention significantly increased participants’ knowledge about STIs and prevention methods at the immediate post-session assessment. Notably, the improvement in knowledge was maintained when reassessed three months later. Knowledge does not necessarily lead to adopting STI/HIV protective behaviors. Still, the improvement in knowledge scores indicates that the intervention successfully conveyed important information to participants, and that the information was retained over time.

Although this pilot study was not powered to detect significant changes in sexual behavior or incidence of STIs, positive signals indicated that the intervention may have altered participants’ behavior. Reported use of both male and female condoms increased in the 3 months after the intervention. Condom use at last sex increased by 10%; a similar absolute increase in reported condom use over the previous 14 days in the HORIZON study resulted in a 35% reduction in incident chlamydia infection over 12 months [16]. Of note, none of the participants who returned for the final study visit had an incident STI, despite an STI prevalence of 17.4% at enrollment.

This pilot study has several limitations. The small number of participants precluded evaluating the most important outcome measures, including condom use and STI incidence. Psychosocial factors that influence sexual risk behavior were not assessed. It was conducted at only one military base; the preliminary findings may not apply to other locations that differ in geography or cultural context. A full evaluation of the KISS intervention will require an adequately powered trial conducted at multiple sites that incorporates measures of psychosocial determinants of behavior. In addition, the intervention itself was heteronormative in design, while 17% of participants self-identified as something other than heterosexual. Future interventions should address the full range of risk experienced by the military population, including homosexual risk behaviors.

The pilot did confirm previous reports that STIs are a common problem among military personnel and other beneficiaries, with almost one-fifth of participants testing positive for N. gonorrhea and/or C trachomatis at study entry. With over 1.3 million active duty personnel, of whom 73% are 18–30 years old, the U.S. military represents a large group of individuals at risk for STIs [5]. From 2011 to 2019, over 350,000 incident STIs were diagnosed among active duty personnel, with significant implications for service members’ availability and ability to perform their duties [10].

In summary, the KISS intervention was feasible to implement in the military setting and was acceptable to the active duty service members and other medical beneficiaries who participated in the pilot project. Further trials are needed to determine if the KISS intervention, or others, effectively decrease STI incidence in active duty personnel and would be appropriate for more widespread scale-up, dissemination, and implementation by the Department of Defense.

## Supplementary Information

**Additional file 1.** Sexual Risk Assessment (SRA).

**Additional file 2. **STI/HIV Knowledge Assessment. 

**Additional file 3. **Feedback Form. 

## Data Availability

All data from this pilot study are publicly available in the Harvard Dataverse at https://doi.org/10.7910/DVN/WXBHRZ

## Declarations

### Disclaimer

The views expressed are those of the authors and should not be construed to represent the positions of the U.S. Army, the Department of Defense, or HJF.
