# Supplementary material for: Feasibility, acceptability, and short-term impact of a brief sexually transmitted infection intervention targeting U.S. Military personnel and family members
Source: BMC Public Health. 2022 Apr 2;22:640. doi: 10.1186/s12889-022-13096-x (PMC8977033; doi:10.1186/s12889-022-13096-x)
Supplement: Supplementary file 1 — Additional file 1. Sexual Risk Assessment (SRA). [file 12889_2022_13096_MOESM1_ESM.pdf]

Sexual Risk Assessment (SRA)

STI: Infections that can pass from one person to another by sexual contact are called sexually transmitted infections (STIs), also known as sexually transmitted diseases (STDs).

Sexual Risk: Some sexual behaviors and practices put us and our partners at higher risk of getting a STI/STD than others.

- The purpose of this survey is to learn about behaviors that put people at risk of getting a STI/STD.
- Our goal is to use this information to help you prevent STIs.
- If any of the items on this survey raise questions about your sexual health, please discuss the topic with your healthcare provider.

➔ You may be embarrassed, but your answers are very important and will help us provide better care to you. **Your answers are completely confidential, so please answer as accurately as you can.**

- The survey contains 10 pages and takes 15 minutes to complete.
- After 15 minutes there will be a 5 minute break, if needed, to finish the survey.
- Make sure your study ID number is on the top of page 3. Please DO NOT put your name anywhere on this survey.

Thank you for completing the Sexual Risk Assessment (SRA), a survey created by the Military HIV Research Program (MHRP). One of MHRP's missions is to protect US troops and the military family from infection, including sexually transmitted infections (STIs) and human immunodeficiency virus (HIV), the virus that causes AIDS.

DEFINITIONS:

*By **"Sex"** we mean intimate physical contact with another person including penetrative vaginal, oral, or anal sex.*

*By "**Oral sex"** we mean you either received or performed **mouth to genital contact**, including penis, vagina, or anus.*

*By "**Vaginal sex**" we mean you engaged in either insertive or receptive **penetration of the vagina with a partner.***

*By "**Anal sex**" we mean you engaged in either insertive or receptive **penetration of the anus with a partner.***

*Please mark "**Single, living with a partner**" even if you are currently deployed or stationed away from your partner, but normally live with your sexual partner and are not married.*

*By **"Spouse"** we mean someone you are legally married to.*

*By "**Regular/main partner**" we mean **someone you would call a girlfriend or boyfriend OR someone you see regularly to have sex with**. This can include someone you are living with, but **NOT** someone you are married to.*

*"**Separated**" means that you are **no longer in a committed relationship with your spouse, but you are not legally divorced**. Separated ONLY applies to people that are no longer in a committed relationship, NOT separated because of a deployment or station assignment re-location only.*

*By **"Occasional"** we mean **someone that you see every once in a while to have sex with but not** someone you are married to, living with, or have a committed relationship with. This can include someone you refer to as a "friend with benefits".*

*By "**One night stand**" we mean **a partner you had sex with or "hooked-up" with one time**, excluding sex workers.*

*By "**Sex worker"** we mean someone who **solicits sex in exchange for money**, drugs, goods, or services.*

STUDY ID# : ___________________________

A. BACKGROUND INFORMATION

1. Today's date (MM DD YY): |__|__| |__|__| |__|__|
2. Year you were born: |__|__||__|__|
3. What is your gender: ❒ Male ❒ Female
4. What is your race/ethnicity? (Mark **one or more** options to indicate how you identify yourself)
   - ❒ White or Caucasian
   - ❒ Black or African American
   - ❒ Hispanic or Latino
   - ❒ Asian (e.g., Asian Indian, Chinese, Japanese, Korean, Vietnamese)
   - ❒ Puerto Rican
   - ❒ Filipino
   - ❒ Native Hawaiian or (non- Filipino) Pacific Islander (e.g., Samoan, Guamanian, Chamorro)
   - ❒ Mexican/Mexican-American/Chicano
   - ❒ Cuban
   - ❒ American Indian or Alaska Native
   - ❒ Other (please specify) ___________________
5. What is your **highest** level of education completed?
   - ❒ Some high school
   - ❒ High school diploma or GED equivalent
   - ❒ Some college
   - ❒ Associates degree
   - ❒ Bachelor's degree (e.g., BS, BA)
   - ❒ Vocational/technical school (other than military)
   - ❒ Some graduate/professional school after college
   - ❒ Completed graduate/professional degree (e.g., MS, PhD, MD)
6. What is your **current** marital status?
   - ❒ Single
   - ❒ In a committed relationship
   - ❒ Married
   - ❒ Separated
   - ❒ Divorced
   - ❒ Widowed
   - ❒ Other: ________________
7. Do you live with your partner? ❒ Yes ❒ No ❒ NA
8. Has your relationship status changed in the last 12 months? ❒ Yes ❒ No

   → *If YES, please describe* ____
9. If you are married, living with a partner, or in a committed relationship, what is the **longest period of time** you have been away from your partner in the **past 3 months?**
   - ❒ **N/A**, I am not in a committed relationship
   - ❒ Less than 2 weeks
   - ❒ 2 weeks - 1 month
   - ❒ 1 - 2 months
   - ❒ 2 - 3 months

   *By **"away"** we mean you traveled away, resided in a different location, or were unable to visit your partner because of military duty or leave.*

Continued on next page

B. MILITARY SERVICE HISTORY

This section is related to your time in the military. If you are a spouse or child dependent (i.e., a medical beneficiary) and you are not in the military, SKIP to Section C.

10. What is your service branch? ❒ Army ❒ National Guard

11. What is your service component? ❒ Active ❒ Reserve

12. What is your **current** rank? ________

13. What year did you enter the service (YYYY)? ____________

14. How many months total have you been away from your home/permanent duty station(s) in the last 2 years ? (This does not include TDY or training exercises) |__|__| times

15. How many times have you been away from your home/permanent duty station(s) (e.g., deployment, TDY/schooling, special missions) for **1 month or more** since entering service? |__|__| times

C. SEXUAL HEALTH

16. Please rank the following sexual health concerns in order of how important they are to you right now. MOST important (#1) to LEAST important (#5).
    ____ Unplanned pregnancy
    ____ Positive HIV test
    ____ Other positive STI test(s)
    ____ Infertility (inability to have a baby)
    ____ Other (e.g., breast/testicular/prostate cancer, epididymitis)
    ____ Other: ____

17. Have you ever taken a sexual health class? ❒ Yes ❒ No
    ➔ *If Yes,* when: ❒ grade school ❒ high school ❒ college ❒ in the military ❒ on my own
    ❒ other: ______________________

18. Are you interested in learning more about safer-sex and sexual health?
    ❒ Yes ❒ No ❒ Maybe ❒ I don't know

19. Where do you go for information when you have a question about your sexual health? **(Mark ALL that apply)**
    ❒ **N/A** I don't have questions
    ❒ My healthcare provider
    ❒ My unit (medic, commander, etc)
    ❒ A trusted personal source (parents, friends, siblings, teachers, etc)
    ❒ Magazines (Men's Health, Women's Health, Cosmo)
    ❒ Online search engine (google, bing, AltaVista, etc)
    ❒ Publicly edited sites (Wikipedia, forums, etc)
    ❒ "Professionally" published sites (WebMD, Center for Disease Control (CDC))
    ❒ From published, professional, peer-reviewed journal articles or books
    ❒ Other: ____

20. Have you **ever** sought **any** type of care for sexual health **OR** other reproductive health care at a:
   - ❒ **N/A**, have not sought testing OR treatment
   - ❒ Military healthcare facility only
   - ❒ Civilian healthcare facility only
   - ❒ Both Military and Civilian healthcare facilities

21. When was the last time you sought care for:

	Less than 6 months ago	Between 6-12 months ago	More than 1 year ago	Never
**Sexual Health care** (ex: screening or treatment for a sexually transmitted infection)				
a. Routine STI or HIV screening (ex: no symptoms or exposure to STI)	❒	❒	❒	❒
b. STI testing and/or treatment (ex: prompted by possible STI exposure, symptoms, or contact from health dept)	❒	❒	❒	❒
**Reproductive Health care** (ex: routine care, pap smear etc.)				
c. Reproductive health care	❒	❒	❒	❒
**Other Healthcare (Specialists, surgery, routine check-up/vaccination)**	❒	❒	❒	❒

D. SEXUALLY TRANSMITTED INFECTIONS (STIS)

22. Have you ever been discouraged or prevented from seeking STI screening or treatment? ❒ Yes ❒ No

23. Have you ever experienced any possible STI symptoms (i.e. painful urination, genital discharge, pain during intercourse, unusual genital swelling, inflammation, growth, odor, or discomfort)?
    ❒ Yes ❒ No

24. Have you had a burning discharge in the **past 3 months**? ❒ **N/A** ❒ Yes ❒ No

25. Were you circumcised ❒ **N/A** ❒ as a child ❒ as an adult ❒ I am not circumcised

26. Has a doctor or health care provider ever said you had any of the following **(Mark ALL that apply)**

	Ever diagnosed?	Symptoms? ☑ **for 'YES'**	0-3 months ago	4-12 months ago	> 1 year ago
a. Gonorrhea (the "Clap")	❒Yes ❒No	❒	❒	❒	❒
b. Chlamydia	❒Yes ❒No	❒	❒	❒	❒
c. Trichomoniasis	❒Yes ❒No	❒	❒	❒	❒
d. Syphilis	❒Yes ❒No	❒	❒	❒	❒
e. Herpes (HSV)	❒Yes ❒No	❒	❒	❒	❒
f. Hepatitis B	❒Yes ❒No	❒	❒	❒	❒
g. Genital warts (HPV)	❒Yes ❒No	❒	❒	❒	❒
h. Anal warts	❒Yes ❒No	❒	❒	❒	❒
i. Pubic lice (Crabs)	❒Yes ❒No	❒	❒	❒	❒
j. HIV/AIDS	❒Yes ❒No	❒	❒	❒	❒
j. Can't remember name of STI/D	❒Yes ❒No	❒	❒	❒	❒
k. Other:	❒Yes ❒No	❒	❒	❒	❒

27. From what **type of partner** do you believe you acquired your **most recent** sexually transmitted infection? **See page 2 for definitions of partner type.**
    - ❒ **NA I have never been diagnosed with an STI (SKIP to Question 30)**
    - ❒ Spouse
    - ❒ Regular partner (non-spouse)
    - ❒ Occasional
    - ❒ One night stand (other than sex workers)
    - ❒ Sex Worker
    - ❒ I don't know

28. Where do you believe you were infected with **your most recent STI/STD?** (city, state, country)

|________________________||__________||________________|
CITY STATE COUNTRY

29. Was this partner a US service member? ❒ Yes ❒ No ❒ I don't know

E. SEXUAL HISTORY

The next set of questions is about your sexual history. *By sex, we mean intimate physical contact with another person including vaginal, oral, or anal sex.* **Please see the definitions on page 2 for each of these sexual activities. Your answers are strictly confidential.**

30. Past sexual partners: ❒ Men ❒ Women ❒ Both ❒ I have never had sex
31. Last sexual contact (approx. date okay): ______________________________
32. Number of lifetime sexual partners: ❒1-10 ❒11-20 ❒ 21-30 ❒31-40 ❒41-50 ❒ 51-60 ❒ 61-70 ❒ 71-80 ❒ 81-90 ❒ 91-100 ❒ 100+
33. How many times did you have sex in the **past month**? ____________
34. How many times did you have sex in the **past 3 months**? __________
35. In the **past 3 months**, did you have vaginal, anal or oral sex **with the partner types listed below**? Please see page 2 for definitions of each partner type. ☑ **for 'YES'**
    ❒ **N/A I have not had sex in the last three months**

	W/ spouse or main partner	W/ an occasional partner	W/ sex worker or one-time partner	Did you use condoms (protection)?		
				Always (100-90%)	Sometimes (>10<100%)	Never (0-10%)
Oral Sex	❒ Yes<br>❒ No	❒ Yes<br>❒ No	❒ Yes<br>❒ No	❒ main partner<br>❒ occasional partner<br>❒ one-time partner	❒ main partner<br>❒ occasional partner<br>❒ one-time partner	❒ main partner<br>❒ occasional partner<br>❒ one-time partner
Vaginal Sex	❒ Yes<br>❒ No	❒ Yes<br>❒ No	❒ Yes<br>❒ No	❒ main partner<br>❒ occasional partner<br>❒ one-time partner	❒ main partner<br>❒ occasional partner<br>❒ one-time partner	❒ main partner<br>❒ occasional partner<br>❒ one-time partner
Anal Sex	❒ Yes<br>❒ No	❒ Yes<br>❒ No	❒ Yes<br>❒ No	❒ main partner<br>❒ occasional partner<br>❒ one-time partner	❒ main partner<br>❒ occasional partner<br>❒ one-time partner	❒ main partner<br>❒ occasional partner<br>❒ one-time partner

36. How many **NEW** sexual partners have you had in the last:
    a. 3 months? ____Male(s) ____ Female(s)
    b. 12 months? ____ Male(s) ____ Female(s)

37. During the **past 3 months** did you engage in vaginal, oral, or anal sex with more than one person at the same time (i.e., group sex)? ❒ Yes ❒ No

38. During the **past 3 months** was a penis, sex toy, or other object inserted in your anus or in your partner's anus (even briefly or accidentally)? ❒ Yes ❒ No

39. Where have you met sexual partners? **(MARK all that apply)**
   - ❒ **N/A**, I do not seek or meet new sexual partners
   - ❒ Work
   - ❒ Through friends
   - ❒ Through sporting events
   - ❒ Through school
   - ❒ Bar/club/ lounge/restaurant/coffee house
   - ❒ Internet (e.g., Grindr, Facebook, Adam4adam, Craigslist, Plenty of Fish, Tinder, etc)
   - ❒ Other (please specify)________________________________

40. How do you self-identify?
   - ❒ Heterosexual or straight
   - ❒ Homosexual or gay
   - ❒ Bisexual
   - ❒ Asexual
   - ❒ Something else: ______________________
   - ❒ Not sure

F. CONDOM USE

The next set of questions asks about how often you or your partner(s) choose to use condoms during sex and your preferences about condoms. *By sex, we mean intimate physical contact with another person including penetrative vaginal, oral, or anal sex.* **Please remember that your answers are strictly confidential.**

41. Have you ever used a male condom? ❒ Yes ❒ No

42. Various forms of barrier protection for STI/HIV prevention are listed below. Mark the boxes next to each listed type if you have carried or used this product in the past 3 months or ever. If you carried AND used a product, mark BOTH boxes.

	In the past 3 months	Ever
	☑ **ALL that apply**	☑ **ALL that apply**
a. finger cot/stall/condom	❒ Carried<br>❒ Used	❒ Carried<br>❒ Used
b. dental/oral dam	❒ Carried<br>❒ Used	❒ Carried<br>❒ Used
c. female condom	❒ Carried<br>❒ Used	❒ Carried<br>❒ Used
d. male condom	❒ Carried<br>❒ Used	❒ Carried<br>❒ Used
e. Other:	❒ Carried<br>❒ Used	❒ Carried<br>❒ Used

43. How many times did you use a condom in the **past month**? ________

44. How many times did you use a condom in the **past 3 months**? ____________

45. Did you use a condom at your last sexual encounter? ❒ Yes ❒ No ❒ I don't know

46. If you did not use a condom at your last sexual encounter, what were your reasons? **(Mark all that apply)**
   ❒ **N/A**, I used condoms/barrier protection
   ❒ Condoms weren't on my mind
   ❒ Forgot to bring a condom
   ❒ Brought a condom but forgot to use it
   ❒ Use birth control
   ❒ Pregnancy is not a concern
   ❒ Trying to have a baby
   ❒ I only have one partner and we are monogamous
   ❒ STIs are not a concern for me
   ❒ I don't like the way condoms feel during sex
   ❒ My partner didn't want to use a condom
   ❒ I was impaired by alcohol during sex
   ❒ I was impaired by drugs during sex❒ I'm not sure I know how to correctly put on a condom
   ❒ Other: ____

47. Are you/your partner using contraception? ❒ Yes ❒ No ❒ I don't know

48. Are you/your partner trying to get pregnant? ❒ Yes ❒ No ❒ I don't know

G. ALCOHOL USE/SUBSTANCE USE

The next set of questions asks you about your use of alcoholic beverages (that is, beer, wine and hard liquor) and substance use. If you have never used alcohol**, SKIP to Section H on the next page.**

49. How often do you have a drink containing alcohol?
    - ❒ Never
    - ❒ Monthly or less
    - ❒ 2 - 4 times a month
    - ❒ 2 - 3 times a week
    - ❒ 4 or more times a week
    - ❒ Weekends only
    - ❒ Daily

50. How many drinks containing alcohol do you have on a typical day when you are drinking?
    ❒ 1 or 2 ❒ 3 or 4 ❒ 5 or 6 ❒ 7 to 9 ❒ 10 or more

51. Have you ever drank until you "blacked out" or you didn't remember what happened?
    ❒ Yes ❒ No

52. In the **past 3 months**, how frequently did you have sex after consuming alcohol?
    - ❒ **N/A,** I do not drink alcohol
    - ❒ Rarely
    - ❒ Occasionally
    - ❒ Always/typically
    - ❒ Don't know/remember
    - ❒ Never in the past 3 months

CONDOM ATTITUDES

Please read each statement and mark one box to indicate how strongly you agree or disagree with each statement.

Reliability and Effectiveness	Strongly Agree	Agree	Neutral	Disagree	Strongly Disagree
1. Condoms are an effective method of birth control.					
2. Condoms are an effective method of preventing the spread of HIV and other sexually transmitted infections.					
3. I think condoms are an excellent means of contraception.					
4. Condoms are unreliable.					
5. Condoms do not offer reliable protection.					
**Pleasure**					
1. The use of condoms can make sex more stimulating.					
2. Condoms ruin the sex act.					
3. Condoms are uncomfortable for both partners.					
4. Condoms are a lot of fun.					
5. Use of a condom is an interruption of foreplay.					
**Identity Stigma**					
1. Men who suggest using a condom are really boring.					
2. If a couple is about to have sex and the man suggests using a condom, it is less likely that they will have sex.					
3. Women think men who use condoms are jerks.					
4. A woman who suggests using a condom does not trust her partner.					
5. People who suggest condom use are a little bit geeky.					
**Embarrassment About Negotiation and Use**					
1. When I suggest using a condom I am almost always embarrassed.					
2. It is really hard to bring up the issue of using condoms to my partner.					
3. It is easy to suggest to my partner that we use a condom.					
4. I'm comfortable talking about condoms with my partner.					
5. I never know what to say when my partner and I need to talk about condoms or other protection.					
**Embarrassment About Purchase**					
1. It is very embarrassing to buy condoms. .					
2. When I need condoms I often dread having to get them.					
3. I don't think that buying condoms is awkward.					
4. It would be embarrassing to be seen buying condoms in a store.					
5. I always feel really uncomfortable when I buy condoms.					

Continued on next page

H. SOCIAL WELLNESS

53. **During the time periods specified,** did you experience any of the following? **(Mark ALL that apply)**

	No	Yes, in the past 3 Months	Yes in the past 4-12 months	Yes, over 1 year ago
a. Depressive symptoms	❒	❒	❒	❒
b. Anxiety	❒	❒	❒	❒
c. Post-traumatic stress disorder (PTSD)	❒	❒	❒	❒
d. Combat Stress Reaction	❒	❒	❒	❒
e. Traumatic Brain Injury (TBI)	❒	❒	❒	❒
f. Homelessness	❒	❒	❒	❒
g. Unplanned pregnancy	❒	❒	❒	❒
h. Domestic abuse/assault	❒	❒	❒	❒
i. Sexual harassment	❒	❒	❒	❒
j. Unwanted sexual contact	❒	❒	❒	❒

Thank you for taking the time to complete this survey.

All information is kept strictly confidential.

We encourage you to talk to your healthcare provider about any questions you have regarding protecting your sexual health and preventing STIs.

If you have any additional comments about the questions or topics covered in this survey, please explain in the space provided below.

____
