# Supplementary material for: Feasibility, acceptability, and short-term impact of a brief sexually transmitted infection intervention targeting U.S. Military personnel and family members
Source: BMC Public Health. 2022 Apr 2;22:640. doi: 10.1186/s12889-022-13096-x (PMC8977033; doi:10.1186/s12889-022-13096-x)
Supplement: Supplementary file 2 — Additional file 2. STI/HIV Knowledge Assessment. [file 12889_2022_13096_MOESM2_ESM.pdf]

Measurement: 1 2 3

STI/HIV Knowledge Assessment

For each statement below, please circle true (T), false (F), or I don't know (DK). If you don't know, please do not guess; instead, please circle DK.	True (T)	False (F)	Don't know (DK)
1. Genital herpes is caused by the same virus as HIV.	T	F	DK
2. There is no cure for chlamydia.	T	F	DK
3. It is easier to get HIV if a person has another sexually transmitted infection (STI).	T	F	DK
4. There is no cure for gonorrhea.	T	F	DK
5. A woman who has genital herpes can pass the infection to her baby during childbirth.	T	F	DK
6. Human papilloma virus (HPV) can cause genital warts.	T	F	DK
7. HPV can lead to cancer in women.	T	F	DK
8. A person must have vaginal sex to get genital warts.	T	F	DK
9. Frequent urinary infections can cause chlamydia.	T	F	DK
10. Soon after infection with HIV a person develops open sores on his or her genitals.	T	F	DK
11. If a person tests positive for an STI, the test can tell how long the person has had the infection.	T	F	DK
12. Using a natural skin condom (lambskin) can protect a person from getting HIV.	T	F	DK
13. The same virus causes HPV and HIV infections.	T	F	DK
14. Sexually transmitted infections can lead to health problems that are usually more serious for men than women.	T	F	DK
15. If a person tests positive for HIV the test can tell how sick the person will become.	T	F	DK
16. A person who has genital herpes must have open sores to give the infection to a sexual partner.	T	F	DK
17. HPV and herpes are sexually transmitted viruses that do not have a cure.	T	F	DK
18. Some STIs cause infertility if they are not treated.	T	F	DK
19. The female condom is safe to use if a person has a latex allergy.	T	F	DK
20. Condoms can be used as a barrier on sex toys to protect from bacteria and STI's.	T	F	DK
21. Silicone lubricant is recommended for anal sex.	T	F	DK
22. A sexually-transmitted infection can cause permanent damage to the body.	T	F	DK

Measurement: 1 2 3

23. Having one STI makes it easier to get another.	T	F	DK
24. STI screening is included as a part of all annual physical exams.	T	F	DK
25. A person will always have discomfort when an STI is doing damage to the body.	T	F	DK
26. Screening for chlamydia and gonorrhea is often done through a urine test.	T	F	DK
27. Using condoms and dental (oral) dams during oral sex acts can protect against cancers of the throat and genitals.	T	F	DK
28. Natural (lambskin) condoms are an effective alternative to latex condoms for STI and pregnancy prevention.	T	F	DK
29. Polyurethane condoms are an effective alternative to latex condoms for STI and pregnancy prevention.	T	F	DK
30. It is safe to use either water-based or oil-based lubricants with latex condoms.	T	F	DK
