# Supplementary material for: Feasibility, acceptability, and short-term impact of a brief sexually transmitted infection intervention targeting U.S. Military personnel and family members
Source: BMC Public Health. 2022 Apr 2;22:640. doi: 10.1186/s12889-022-13096-x (PMC8977033; doi:10.1186/s12889-022-13096-x)
Supplement: Supplementary file 3 — Additional file 3. Feedback Form. [file 12889_2022_13096_MOESM3_ESM.pdf]

Date: ______________________

Your feedback is very important. You received a lot of information today.
We want to find out if you're really ready to K.I.S.S.

Please rate how much you agree or disagree with the following statements about today's class so we know what was helpful for you and what wasn't.

		Strongly Disagree (1)	Disagree (2)	Neutral (3)	Agree (4)	Strongly Agree (5)
1.	I know what K.I.S.S. stands for.					
2.	I learned a lot from group discussions.					
3.	My moderator explained information clearly.					
4.	I feel more confident talking about STIs.					
5.	I felt comfortable asking questions.					
6.	I know how to prevent STIs using safe sex products.					
7.	I am more likely to practice safe sex after today's class.					
8.	I felt comfortable with my group today.					
9.	I will get screened regularly for STIs.					
10.	I understand what 'safe sex' means.					
11.	I know the difference between STI and pregnancy prevention.					
12.	I will recommend this class to a friend.					

What did you learn today?

1. ______________________________
2. ______________________________
3. ______________________________

What did you hope to learn, but didn't?

1. ______________________________
2. ______________________________

What will you do differently after today's class?

1. ______________________________
2. ______________________________

Thank you for your participation!
